# Direct serogrouping of *Dichelobacter nodosus* from Victorian farms using conventional multiplex polymerase chain reaction

**DOI:** 10.1186/s13104-018-3229-5

**Published:** 2018-02-07

**Authors:** Nickala Best, Jacek Gwozdz, Robert Suter, Grant Rawlin, Travis Beddoe

**Affiliations:** 1 Department of Animal, Plant and Soil Science and Centre for AgriBioscience (AgriBio), La Trobe University, Bundoora, Melbourne, VIC Australia; 2 Department of Economic Development, Jobs, Transport and Resources Centre for AgriBioscience (AgriBio), Victorian Government, Bundoora, Melbourne, VIC Australia; 3 Department of Economic Development, Jobs, Transport and Resources, Victorian Government, Attwood, Melbourne, VIC Australia

**Keywords:** Footrot, Serogroup, PCR

## Abstract

**Objective:**

*Dichelobacter nodosus* is the causative agent of footrot in sheep. Ovine footrot is a major problem in Australia that results in large economic losses and a represents a very significant animal welfare issue. *D. nodosus* is divided into 10 serogroups (A–I, M), based on sequence variation in the type IV fimbriae gene, *fimA*. Control of the bacteria is possible through use of serogroup-specific vaccination, however traditional identification of the serogroups of *D. nodosus* on infected sheep is time-consuming and costly. With the aim of reducing time and cost, a PCR assay was used to identify serogroups of *D. nodosus* directly from foot swabs of infected sheep in Victoria.

**Results:**

It was shown that serogroup B was most common (10 locations), followed by A, G and H (4 locations), I and C (2 locations), D, E and F (1 location). Infections with multiple serotypes were observed in 50% of farms, with the remaining 50% having only a single serogroup detected. The ability to identify serogroups quickly and cheaply direct from foot swabs will aid the understanding of the epidemiology of *D. nodosus* and support control programs.

**Electronic supplementary material:**

The online version of this article (10.1186/s13104-018-3229-5) contains supplementary material, which is available to authorized users.

## Introduction

Ovine footrot is a bacterial infection in sheep, with virulent infections showing severe lesions in the hoof and resulting in lameness [1]. Footrot infections cost the sheep meat and wool industry $45 million per year in Australia [2]. The causative agent, *Dichelobacter nodosus*, is a genetically diverse, gram-negative anaerobe. *D. nodosus* is classified into different serogroups based on the sequence of the fimbrial A gene (*fimA).* In Australia, the bacteria are classed into 10 serogroups (A–I, M), with serogroups A–I being fully sequenced, while the Australian serogroup M has been partially sequenced [3]. Each serogroup elicits a specific and strong immune response from the host. Vaccination with serogroup-specific FimA antigen provides a protective response, however, cross protection between serogroups does not occur, and multiple serogroup infections have been reported to be common [4]. Understanding what *D. nodosus* serogroups are on a particular farm can support better epidemiological investigations, control and eradication programs [5]. Currently, the testing for serotype of *D. nodosus* is time-consuming and labour intensive. It requires bacterial isolation, culturing and slide agglutination testing to determine the serogroup. Slide agglutination requires the production antisera specific for each serogroup, which is beyond most diagnostic laboratories capacity.

Recently, a multiplex PCR was developed to determine the serogroup of *D. nodosus* from bacterial colonies grown on media [6]. This molecular assay has been shown to be highly specific and reduces time in identifying the serogroup of *D. nodosus*. The ability to determine *D. nodosus* serogroup directly from foot swabs would aid epidemiological investigations of footrot in Australia. Here, we have identified the serogroups of *D. nodosus* from foot swabs collected on Victorian farms using this multiplex PCR assay.

## Main text

### Materials and methods

#### Field collection

**Fig. 1:**
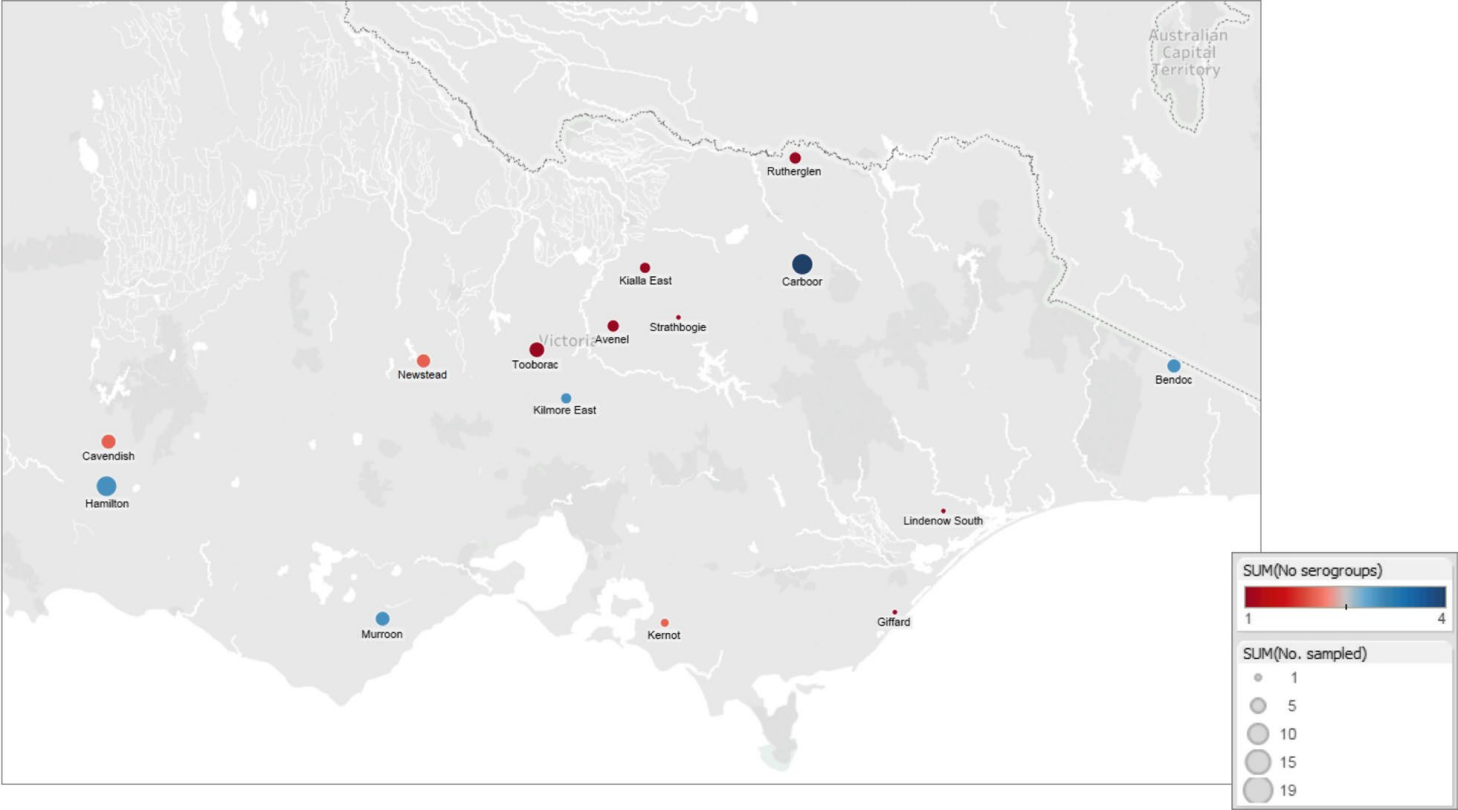
Victorian farms sampled. The locations of the 16 Victorian farms sampled and number of serogroups detected at each location. The figure was generated in Tableau 6.1

The clinical samples were obtained retrospectively from the Victorian Government Veterinary Diagnostic unit collection. The original samples were submitted by Victorian District Veterinary Officers, Animal Health Officers and private veterinary practitioners from the interdigital skin of lame sheep for routine diagnostic testing. Samples (n = 137) were collected from 16 farms across 15 Victorian locations. These farms were diagnosed as clinically virulent, benign or healthy during a disease surveillance survey from November 2014 to August 2015 (Fig. 1). The interdigital skin was swabbed and the cotton head of the swab transported at 4 °C in phosphate buffer saline (PBS) (8.1 mM Na_2_HPO_4_, 137 mM NaCl, 1.4 mM KH_2_HPO_4_ and 2.6 mM KCl) with an addition of 20 mM ethylenediaminetetraacetic acid (EDTA). Total nucleic acid was extracted using the MagMAX™ − 96 Viral RNA isolation kit (Ambion, Austin, USA) and Kingfisher-96 magnetic particle handling system (Thermo Fisher Scientific, Finland), as per manufacturer’s instructions.

#### qPCR for detection of *D. nodosus*

Detection of *D. nodosus* was performed by quantitative polymerase chain reaction (qPCR) as described by Stäuble et al. [7]. Briefly, the qPCR program consisted of an initial denaturation step of 2 min at 50 °C, a hold of 10 min at 95 °C, followed by 40 cycles of 15 s at 95 °C, 1 min at 60 °C. The AgPath-IDTM One-Step RT-PCR Kit (Ambion, Austin, USA) was used as master mix according to manufacturer’s instructions, with final concentrations of 300 nM primers, 100 nM DnAprTM- vMGB, 250 nM DnAprTM-bMGB and 5 μL of template DNA used. Primers and probes were synthesised and supplied by Applied Biosystems (California, USA). Reactions were carried out and analysed using the 7500 Fast Real-Time PCR System (Life Technologies), with a set threshold of 0.05. Results are reported as cycling threshold (Ct) values, the point at which the sample signal exceeds the threshold of 0.05.

#### Serotyping by PCR

Multiplex PCR for determination of serogroup was performed as described by Dhungyel et al. with the following modifications [6]. GoTaq^®^ Green Master Mix 2X (Promega, Madison, USA) was used for amplification as per manufacturer’s instructions, with the addition of 1 µL 100 mM MgSO_4_ to increase the Mg^+^ concentration to 5.5 mM. Reactions were carried out in a final volume of 25 µL and amplified in a C-Master GT thermal cycler (Dynamica, Australia). The following amplification conditions consisted of an initial denaturation step of 4 min at 95 °C, followed by 5 cycles of 30 s at 94 °C, 30 s at 60 °C, and 30 s at 72 °C, followed by 25 cycles of 30 s at 94 °C, 30 s at 58 °C, 30 s at 72 °C for 30 s and then a final elongation of 4 min at 72 °C. Amplicon size was checked by agarose electrophoresis migration by loading directly on 2% (w/v) MetaPhor^®^ Agarose (Lonza, USA) gel, prepared as per manufacturer’s instructions, with the addition of 0.5 µL of Sybr^®^ Safe DNA Gel Stain (Life Technologies). The gel was run at 100 V for 55 min and imaged using the GelDoc™ XR+ (BioRad) instrument and software. Sanger DNA sequencing of representative amplicons was performed at Australian Genomics Research Facility (AGRF) to verify amplified serogroups.

### Results

**Fig. 2:**
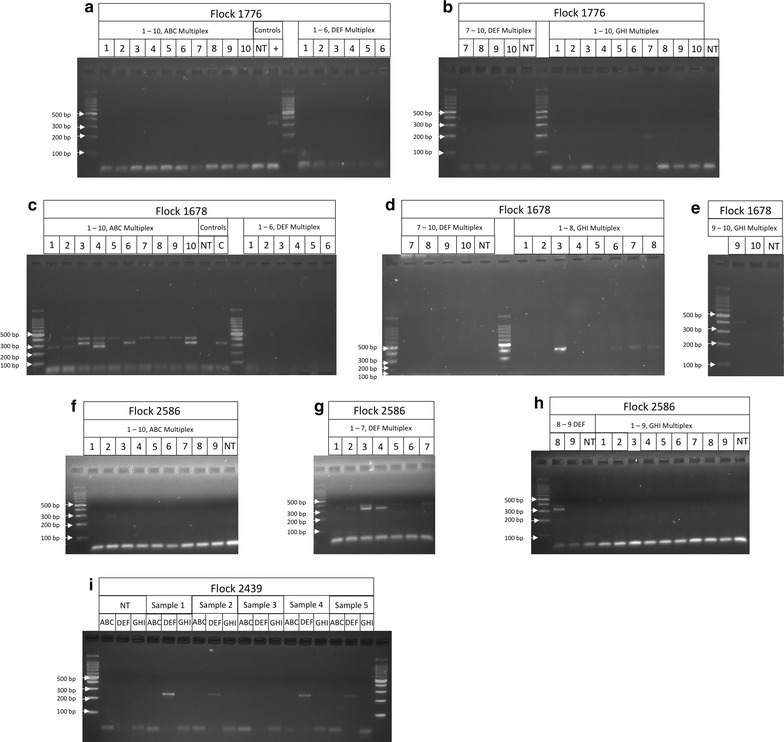
Identification of serogroups from foot swab samples by PCR. Samples from various farms were amplified in serogroups mixtures (ABC, DEF, and GHI) and analyzed on a 2% MetaPhor agarose gel. Banding is shown that corresponds to serogroup I (**a** and **b**), serogroups A, B, C and H (**c**, **d** and **e**), serogroups B, D and E (**f**, **g** and **h**) and serogroup F (**i**). The serogroup A and C positive control with no amplification seen in the negative, no template (NT), control

Foot swab samples were collected from farms that have been known previously to have clinical forms of footrot, or were clinically healthy yet had *D. nodosus* detected on animals. These samples and the collection method were used due to availability, as they were concurrently used for additional studies. Genomic DNA was extracted from all samples and the presence of *D. nodosus* in all 137 sheep were confirmed by qPCR as described by Stäuble et al. [7]. Initial experiments to determine the serotype of *D. nodosus* by PCR from DNA extracted from foot swabs was unsuccessful, however the addition of extra magnesium to the PCR reaction was able to achieve amplification of PCR products for the determination of serogroups present on different farms (Fig. 2). The representative PCR products were verified by DNA sequencing as shown in Additional file 1: Figure S1.

Overall the result revealed that 87 (63%) of the sheep tested resulted in a serogroup being determined by multiplex PCR using DNA directly extracted from a foot swab sample (Table 1). From the animals that showed a serogroup band, 74% of the infected sheep had a single serogroup detected, while 26% had infections from multiple serogroups, ranging from 2 to 4 serogroups detected (Table 1). Serogroup B was the most commonly detected serogroup as shown in Additional file 2: Figure S2), found in 48% of infections, on 56% of farms and in 60% of locations in Victoria. Serogroup B was also involved in 91% of the farms with multiple serogroup infections. Serogroups A, G and H were the next most common serogroups, found in 15, 25 and 12% of infections respectively. All were found in 4 locations in both single and multiple infections. Serogroup C and I were found in 2 farms in 2 separate locations, with D, E and F present in 1 location only.

**Table 1:** Summary of serogroups found on farms across different locations in Victoria

Location	Farms	Animals tested (n)	Animals with serogroup detected (n)	Serogroup (times detected)	Animals with multiple serogroups (n)
Avenel	1	10	6	G (6)	0
Bendoc	1	10	4	G (4), H (2)	2
Carboor	1	10	9	A (7), B (3), C (4), H (5)	8
Cavendish	1	10	9	B (1), G (8)	5
Giffard	1	1	1	C (1)	0
Hamilton	2	20	15	B (8), G (3), F (7)	3
Kernot	1	2	2	A (2), H (1)	1
Kialla East	1	10	5	I (5)	0
Kilmore East	1	10	4	A (1), B (2), H (2)	1
Lindenow South	1	10	1	I (1)	0
Murroon	1	9	6	B (1), D (4), E (4)	3
Newstead	1	10	8	A (3), B (5)	0
Rutherglen	1	9	6	B (6)	0
Strathbogie	1	6	1	B (1)	0
Tooborac	1	10	10	B (10)	0
Total	16	137	87		23

The animals tested were all confirmed to be *D. nodosus* positive via qPCR

### Discussion

We have modified an existing protocol by increasing the concentration of MgCl_2_, for faster and more cost-effective identification of *D. nodosus* serogroup determination by PCR, by analysing DNA extracted from foot swabs. This has allowed us to investigate the epidemiology of *D. nodosus* infection on farms throughout Victoria. It was found that on the sampled farms, there was a high level of detection of serogroup B. This is in agreement with a previous study that showed serogroup B was most commonly detected [8]. Additionally, it was also found that serogroup B was most commonly found in multiple infections.

Additional sampling across more locations is needed to gain an overall insight into the prevalence of different serogroups in Victoria, with this method providing a starting point for a higher throughput study. Currently, Victorian diagnostics laboratories are not equipped to isolate or grow *D. nodosus* or produce the antisera that is required for the traditional slide agglutination tests to determine serogroup. The further improvement of the methods here would allow for molecular diagnostics based laboratories to access this information. A more affordable and accessible method of serotyping would aid in epidemiological studies in Victoria and contribute to the improved control of the disease.

These results give a basic overview of the epidemiology of *D. nodosus* on farms throughout Victoria, which previously has not been known. The ability to serotype directly from a swab greatly reduces the time and cost traditionally required to serotype field samples. This information and additional virulence testing may help in both understanding and control of the disease in the future.

## Limitations

It should be noted that not all samples tested returned a visible serogroup PCR band, and these will require further investigation. Experimentally, it may be possible bands were present yet were not detected using the current method. Additional detection methods were not investigated. There is also the possibility that undetermined samples belong to serogroup M as this was not included in the PCR. There is limited information on the prevalence of serogroup M in Australian isolates, however, in two studies the numbers ranged from 5 to 40% of flocks [3, 9]. Currently, serogroup M identification requires bacterial isolation, culturing and slide agglutination due to the lack of suitable primers for identification of serogroup M by PCR. Therefore, it is imperative to develop primers suitable for detection of serogroup M by PCR to understand the prevalence of serogroup M in Victorian sheep flocks.

## Additional files

**Additional file 1: Figure S1.** PCR amplicons of different serogroups subjected to Sanger sequencing. Blastn alignment of serogroup G (panel A), I (panel B), B (panel C, E, F and G) and F (panel D) amplicon sequence obtained from Sanger sequencing from various farms. Accession number is shown in brackets.

**Additional file 2: Figure S2.** PCR amplicons of different serogroups subjected to Sanger sequencing. Blastn alignment of serogroup G (panel A), I (panel B), B (panel C, E, F and G) and F (panel D) amplicon sequence obtained from Sanger sequencing from various farms. Accession number is shown in brackets.

## Abbreviations

qPCR: quantitative polymerase chain reaction
EDTA: ethylenediaminetetraacetic acid
DNA: deoxyribonucleic acid
PBS: phosphate buffer saline
