# Supplementary material for: Direct serogrouping of Dichelobacter nodosus from Victorian farms using conventional multiplex polymerase chain reaction
Source: BMC Res Notes. 2018 Feb 7;11:108. doi: 10.1186/s13104-018-3229-5 (PMC5804069; doi:10.1186/s13104-018-3229-5)
Supplement: Supplementary file 1 — Additional file 1: Figure S1. PCR amplicons of different serogroups subjected to Sanger sequencing. Blastn alignment of serogroup G (panel A), I (panel B), B (panel C, E, F and G) and F (panel D) amplicon sequence obtained from Sanger sequencing from various farms. Accession number is shown in brackets. [file 13104_2018_3229_MOESM1_ESM.pdf]

A

```
Flock 2177              1   AGAGCGTACTTACCCATATCTGTGTTACCTACTTCGCCAGATGCAGGATCTCCTTCAGAG  60
                            ||||||||||||||||||||||||||||||||||||||||||||||||||||||||||||
Serogroup G(Y00425)629      AGAGCGTACTTACCCATATCTGTGTTACCTACTTCGCCAGATGCAGGATCTCCTTCAGAG  570

Flock 2177             61   GTACATTTACCATCTTGTAAGTTGTCAGCGATACGGATTTTTAAACCATCAGCCAAGCTA  120
                            ||||||||||||||||||||||||||||||||||||||||||||||||||||||||||||
Serogroup G(Y00425)569      GTACATTTACCATCTTGTAAGTTGTCAGCGATACGGATTTTTAAACCATCAGCCAAGCTA  510

Flock 2177            121   ACGCCTTCTGAAACCTGGGTACGAGCGATGTAGTCGTTGTATGCAGGGATAGCGAAAGCA  180
                            ||||||||||||||||||||||||||||||||||||||||||||||||||||||||||||
Serogroup G(Y00425)509      ACGCCTTCTGAAACCTGGGTACGAGCGATGTAGTCGTTGTATGCAGGGATAGCGAAAGCA  450

Flock 2177            181   GCTAAGATACCGATAATTGCAACCACAATCATGAGTTCGATTAAGG  226
                            ||||||||||||||||||||||||||||||||||||||||||||||
Serogroup G(Y00425)449      GCTAAGATACCGATAATTGCAACCACAATCATGAGTTCGATTAAGG  404
```

B

```
Flock 2178                      1 CGATGCGGATTTTTAA-CCATCAGCCAAACTAACGCCTTCAGCTGCTTGTGAACGAGCGA  59
                                  |||||||||||||||| |||||||||||||||||||||||||||||||||||||||||||
Serogroup I (KR861701.1) 196 CGATGCGGATTTTTAAACCATCAGCCAAACTAACGCCTTCAGCTGCTTGTGAACGAGCGA  137

Flock 2178                     60 TGTAGTCGTTGTATGCAGGGATAGCGAAAGCCGCTAAGATACCGATAATTGCAACTACAA  119
                                  ||||||||||||||||||||||||||||||||||||||||||||||||||||||||||||
Serogroup I (KR861701.1) 136 TGTAGTCGTTGTATGCAGGGATAGCGAAAGCCGCTAAGATACCGATAATTGCAACTACAA  77

Flock 2178                    120 TCATGAGTTCGATTAAGG  137
                                  ||||||||||||||||||
Serogroup I (KR861701.1)  76 TCATGAGTTCGATTAAGG  59
```

C

```
Flock 2183                    1 TGCT-AGGCATACTTACCTTTGTCTTCGTTGCCAACAACACCAGATTGTGGATCTGCGTC 59
                                |||| |||||||||||||||||||||||||||||||||||||||||||||||||||||||
Serogroup B(KT831901.1)  278 TGCTAAGGCATACTTACCTTTGTCTTCGTTGCCAACAACACCAGATTGTGGATCTGCGTC 219

Flock 2183                   60 CGGTCCTTTACATTCGCCGTCTTGTAAGTTTTCAGCGATACGAACTTTCAAACCATCAGC 119
                                ||||||||||||||||||||||||||||||||||||||||||||||||||||||||||||
Serogroup B(KT831901.1)  218 CGGTCCTTTACATTCGCCGTCTTGTAAGTTTTCAGCGATACGAACTTTCAAACCATCAGC 159

Flock 2183                  120 CAAGCTTACGCCTTCAGCTGCTTGTGAACGAGCGATGTAGTCGTTGTATGCAGGGATAGC 179
                                ||||||||||||||||||||||||||||||||||||||||||||||||||||||||||||
S Serogroup B(KT831901.1) 158 CAAGCTTACGCCTTCAGCTGCTTGTGAACGAGCGATGTAGTCGTTGTATGCAGGGATAGC 99

Flock 2183                  180 GAAAGCCGCTAAGATACCGATAATTGCAACTACAATCATGAGTTCGATTAAGG  232
                                |||||| ||||||||||||||||||||||||||||||||||||||||||||||
Serogroup B(KT831901.1)    98 GAAAGCAGCTAAGATACCGATAATTGCAACTACAATCATGAGTTCGATTAAGG  46
```

D

```
Flock 2349                  1   GCACCAGCATTAGCAGTTTCCATACAAGAGCCATTTTCTAAGTGATCAGCGATACGGATT   60
                                ||||||||||||||||||||||||||||||||||||||||||||||||||||||||||||
Serogroup F (X52408.1) 245   GCACCAGCATTAGCAGTTTCCATACAAGAGCCATTTTCTAAGTGATCAGCGATACGGATT  186

Flock 2349                 61   TTTAAACCATCAGCCAATGTTAAGCCTTCAGCTGCTTGTGAACGAGCGATGTAGTCGTTG  120
                                ||||||||||||||||||||||||||||||||||||||||||||||||||||||||||||
Serogroup F (X52408.1) 185   TTTAAACCATCAGCCAATGTTAAGCCTTCAGCTGCTTGTGAACGAGCGATGTAGTCGTTG  126

Flock 2349                121   TATGCAGGGATAGCGAAAGCCGCTAAGATACCGATAATTGCAACTACAATCATGAGTTCG  180
                                ||||||||||||||||||||||||||||||||||||||||||||||||||||||||||||
Serogroup F (X52408.1) 125   TATGCAGGGATAGCGAAAGCCGCTAAGATACCGATAATTGCAACTACAATCATGAGTTCG  66

Flock 2349                181   ATTA  184
                                ||||
Serogroup F (X52408.1)  65   ATTA  62
```

E

```
Flock 2234               1  GCT-AGGCATACTTACCTGTGTCTTTGTTGCCAACAACACCAGATGCTGGATCTGCGTCC  59
                            ||| ||||||||||||||||||||||||||||||||||||||||||||||||||||||||
Serogroup B(M37471.1)  287  GCTAAGGCATACTTACCTGTGTCTTTGTTGCCAACAACACCAGATGCTGGATCTGCGTCC  228

Flock 2234              60  GGTCCTTTACATTCGCCGTCTTGTAAGTTTTCAGCGATACGAACTTTCAAACCATCAGCC  119
                            ||||||||||||||||||||||||||||||||||||||||||||||||||||||||||||
Serogroup B(M37471.1)  227  GGTCCTTTACATTCGCCGTCTTGTAAGTTTTCAGCGATACGAACTTTCAAACCATCAGCC  168

Flock 2234             120  AAGCTTACGCCTTCAGCTGCTTGTGAACGAGCGATGTAGTCGTTGTATGCAGGGATAGCG  179
                            |||||||||||||||||||||||||||||||||||||||||||| |||||||||||||||
Serogroup B(M37471.1)  167  AAGCTTACGCCTTCAGCTGCTTGTGAACGAGCGATGTAGTCGTTATATGCAGGGATAGCG  108

Flock 2234             180  AAAGCCGCTAAGATACCGATAATTGCAACTACAATCATGAGTTCGATTAAGG  231
                            ||||||||||||||||||||||||||||||||||||||||||||||||||||
Serogroup B(M37471.1)  107  AAAGCCGCTAAGATACCGATAATTGCAACTACAATCATGAGTTCGATTAAGG  56
```

F

```
Flock 2436               1  TGCT-AGGCATACTTACCTTTGTCTTGGTTGCCAACAACACCAGATCCTGGATCTGCGTC  59
                            |||| ||||||||||||||||||||| ||||||||||||||||||| |||||||||||||
Serogroup B(M92190.1)  617  TGCTAAGGCATACTTACCTTTGTCTTCGTTGCCAACAACACCAGATGCTGGATCTGCGTC  558

Flock 2436              60  CGGTCCTTTACATTCGCCGTCTTGTAAGTTTTCAGCGATACGAACTTTCAAACCATCAGC  119
                            ||||||||||||||||||||||||||||||||||||||||||||||||||||||||||||
Serogroup B(M92190.1)  557  CGGTCCTTTACATTCGCCGTCTTGTAAGTTTTCAGCGATACGAACTTTCAAACCATCAGC  498

Flock 2436             120  CAAGCTTACGCCTTCAGCTGCTTGTGAACGAGCGATGTAGTCGTTGTATGCAGGGATAGC  179
                            ||||||||||||||||||||||||||||||||||||||||||||||||||||||||||||
Serogroup B(M92190.1)  497  CAAGCTTACGCCTTCAGCTGCTTGTGAACGAGCGATGTAGTCGTTGTATGCAGGGATAGC  438

Flock 2436             180  GAAAGCCGCTAAGATACCGATAATTGCAACTACAATCATGAGTTCGATTA  229
                            ||||||||||||||||||||||||||||||||||||||||||||||||||
Serogroup B(M92190.1)  437  GAAAGCCGCTAAGATACCGATAATTGCAACTACAATCATGAGTTCGATTA  388
```

G

```
Flock 2519               1  GCT-AGCCATACTTACCTTTGTCTTCGTTGCCAACAACACCAGATGCTGGATTTGCGTCC  59
                            ||| ||||||||||||||||||||||||||||||||||||||||||||| ||||||||||
Serogroup B(X52404.1)  366  GCTAAGCCATACTTACCTTTGTCTTCGTTGCCAACAACACCAGATGCTGTATTTGCGTCC  307

Flock 2519              60  GGTCCTTTACATTCGCCGTCTTGTAAGTTTTCAGCGATACGAACTTTCAAACCATCAGCC  119
                            ||||||||||||||||||||||||||||||||||||||||||||||||||||||||||||
Serogroup B(X52404.1)  306  GGTCCTTTACATTCGCCGTCTTGTAAGTTTTCAGCGATACGAACTTTCAAACCATCAGCC  247

Flock 2519             120  AAGCTTACGCCTTCAGCTGCTTGTGAACGAGCGATGTAGTCGTTATATGCAGGGATAGCG  179
                            |||||||||||||||||||||||||||||||||||||||||||| |||||||||||||||
Serogroup B(X52404.1)  246  AAGCTTACGCCTTCAGCTGCTTGTGAACGAGCGATGTAGTCGTTGTATGCAGGGATAGCG  187

Flock 2519             180  AAAGCAGCTAAGATACCGATAATTGCAACTACAATCATGAGTTCGATTAAGG  231
                            ||||| ||||||||||||||||||||||||||||||||||||||||||||||
Serogroup B(X52404.1)  186  AAAGCCGCTAAGATACCGATAATTGCAACTACAATCATGAGTTCGATTAAGG  135
```
