# Supplementary figures and images for: Direct serogrouping of Dichelobacter nodosus from Victorian farms using conventional multiplex polymerase chain reaction
Source: BMC Res Notes. 2018 Feb 7;11:108. doi: 10.1186/s13104-018-3229-5 (PMC5804069; doi:10.1186/s13104-018-3229-5)

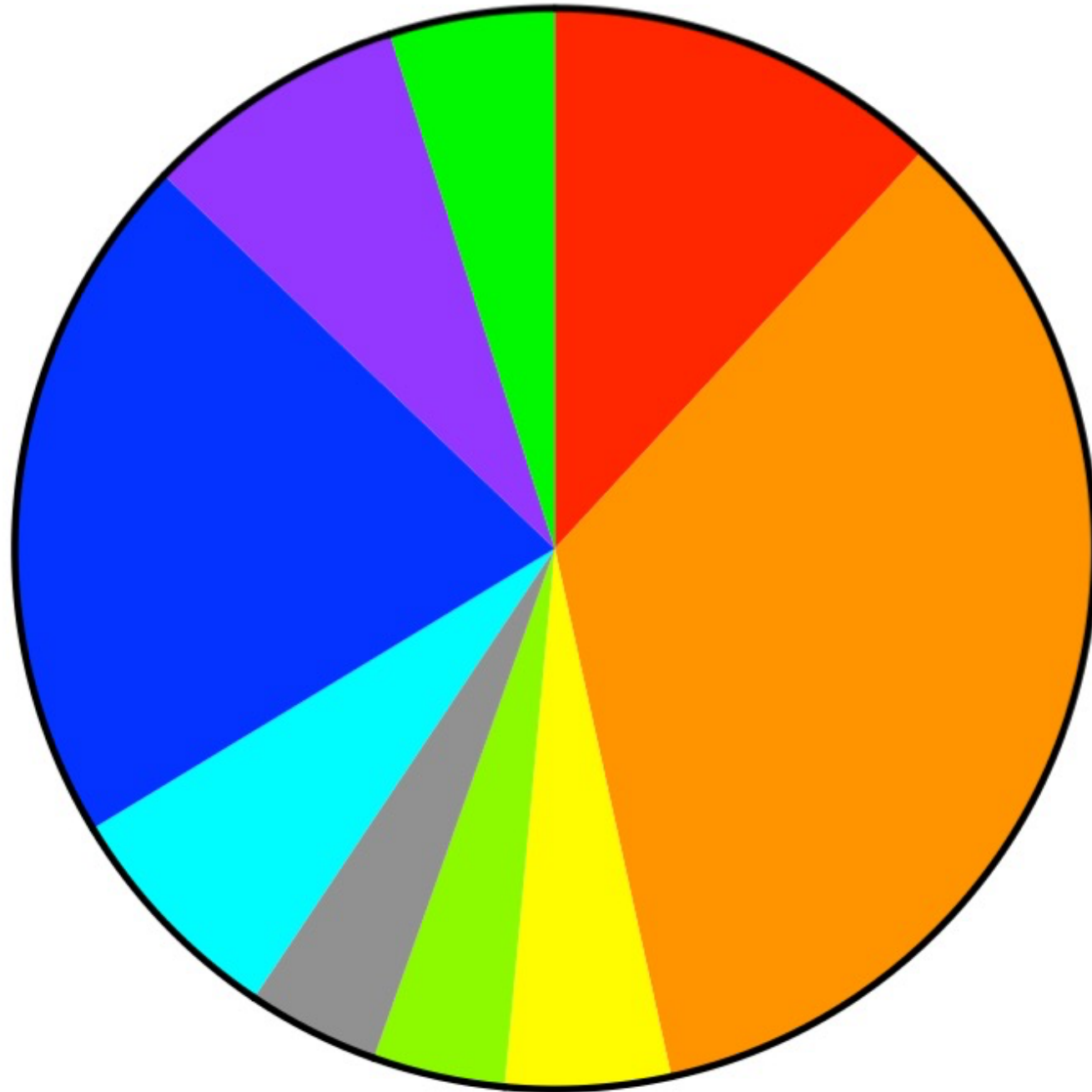

- A (n=12)
- B (n=35)
- C (n=5)
- D (n=4)
- E (n=4)
- F (n=7)
- G (n= 21)
- H (n=8)
- I (n=5)

Supplement: Supplementary file 2 — Additional file 2: Figure S2. PCR amplicons of different serogroups subjected to Sanger sequencing. Blastn alignment of serogroup G (panel A), I (panel B), B (panel C, E, F and G) and F (panel D) amplicon sequence obtained from Sanger sequencing from various farms. Accession number is shown in brackets. [file 13104_2018_3229_MOESM2_ESM.pdf]
